# Dietary specialization depending on ecological context and sexual differences in Asiatic black bears

**DOI:** 10.1371/journal.pone.0223911

**Published:** 2019-10-18

**Authors:** Tomoki Mori, Saki Nakata, Shigeyuki Izumiyama

**Affiliations:** 1 Interdisciplinary Graduate School of Science and Technology, Shinshu University, Minamiminowa, Kamiina-gun, Nagano Prefecture, Japan; 2 Graduate School of Science and Technology, Shinshu University, Minamiminowa, Kamiina-gun, Nagano Prefecture, Japan; 3 Institute of Mountain Science, Shinshu University, Minamiminowa, Kamiina-gun, Nagano Prefecture, Japan; Universidad Nacional Autonoma de Mexico Instituto de Investigaciones en Ecosistemas y Sustentabilidad, MEXICO

## Abstract

The food habits of the Asiatic black bear (*Ursus thibetanus*) are well studied, but there is a little evidence of dietary specialization—that is, when individuals use a narrower set of resources compared to the population as a whole. To examine the dietary composition at the individual level, seasonal patterns of dietary specialization, and sex-based dietary differences in Asiatic black bears, we attached Global Positioning System (GPS) collars to 15 Asiatic black bears and collected their scats in Nagano Prefecture, Japan from 2017 to 2018. Our results showed that the dietary composition differed among individuals, although seasonal changes in dietary composition were observed at the population level. Dietary specialization was high in summer (resources less abundant) and low in spring and autumn (resources more abundant), indicating a relationship with general food abundance and the dietary diversity of bears. In spring, all bears consumed green vegetation and/or seed of Fagaceae family from previous autumn; in early- and late- summer, dietary composition, such as green vegetation, insects, and fruits, greatly differed among individuals. In autumn, most bears heavily depended on seeds of Fagaceae which is high-quality food for bears.

Although we did not find statistical differences between sexes in terms of dietary specialization and diversity, we found variations in the timing of feeding on the Fagaceae family, being earlier in females compared with males. We also found considerable variation in dietary composition within sexes, suggesting that dietary specialization depends on multiple factors besides food abundance, food diversity, and sex.

## Introduction

A generalist species uses a broad range of habitats and food resources, thereby thriving in a wide variety of environmental conditions. However, many populations of generalist species actually comprise individual specialists—that is, a group of individuals specializing in a narrow array of different food types [1]. Such dietary specialization can mitigate inter- and intraspecific competition, resulting in long-term stability at the population level [2]. Therefore, dietary specialization is considered an important factor in population dynamics and conservation ecology [2].

Dietary specialization is believed to arise from interactions between individuals’ intrinsic traits and ecological contexts [3,4]. Recent frame works of nutrient and nutritional geometry has shown that many animal species maximize energy intake by selecting mixed diets and adjusting the intake of macronutrients (proteins, carbohydrates, and lipids), instead of eating specific high-energy diet [5]. Because optimal nutritional and energy intake varies with sex- or age, each individual selects different foods to meet their own nutritional requirements [6], resulting in sex- and age- specific differences in foraging strategies (i.e., dietary specialization). Furthermore, food abundance and diversity relate to the level of intraspecific competition and dietary specialization, such as diet partitioning between dominant and sub-dominant individuals [7]. Despite the fact that dietary specialization is important to understanding competitive interactions and population dynamics, its implications, particularly for terrestrial mammals, are still unclear.

Asiatic black bears (*Ursus thibetanus*) inhabiting Japan are omnivorous and mainly consume plant-based materials [8]. Therefore, depending on plant phenology, they forage for a broad range of food sources: in spring, green vegetation, such as new leaves and flowers; in summer, green vegetation, insects, and fleshy fruits; and in autumn, seeds of the Fagaceae family [9–12]. Although the Asiatic black bears’ seasonal food habits are comparatively well studied, there is little evidence of dietary specialization [13]. In general, food abundance changes seasonally, being highest in autumn (when abundant fruits are found), lowest in summer, and medium in spring [14]. This idea is strongly supported by the fact that the body condition of Asiatic black bears in Japan continuously deteriorates from spring to summer and subsequently recovers [15,16]. Therefore, because of the fluctuation in food abundance and the related food habits of Asiatic black bears, their dietary specialization may also change seasonally. In addition, most bear species are sexually dimorphic, with males being typically larger than females [17–19] and females requiring extra energy compared to males, such as during pregnancy and lactation [20]. Therefore, sex might be an important driver of dietary specialization in Asiatic black bears.

In this study, we used Global Positioning System (GPS) to conduct individual level diet surveys of Asiatic black bears by collecting their scats at GPS clusters via remotely collected location data. We described seasonal patterns of dietary specialization, dietary composition at the individual level, and sex-specific dietary differences in the bears in two different years. First, we predicted that dietary specialization is high in summer when there is a relatively low abundance of food, and low in spring and autumn when the abundance of food is relatively moderate or high, respectively because food scarce environment would lead to partitioning of foraging strategy in relation to intraspecific competition in addition to sex- and age-based differences. Second, we predicted that sex is a driver of dietary specialization in relation to physiological requirements. Third, we predicted that the timing of Fagaceae feeding occurs earlier for female bears, because female need to perform parenting which requires them to accumulate more fat than male bears before hibernation.

## Methods

### Ethics statements

Our research did not involve endangered species. All procedures for the capture and handling protocol of Asiatic black bears were followed based on the guidelines established by the Mammal Society of Japan (2009). We obtained permission from the Nagano Prefectural Office for capture of bears. No permissions were required for the locations of bear capturing because hunters have permission from Ina city office instead. In addition, we obtained consent from Ina city office, Kiso town hall, Komagane town hall, and the land owner for location of scat sampling in the field (public forest lands and private forest).

### Study area

**Fig 1:**
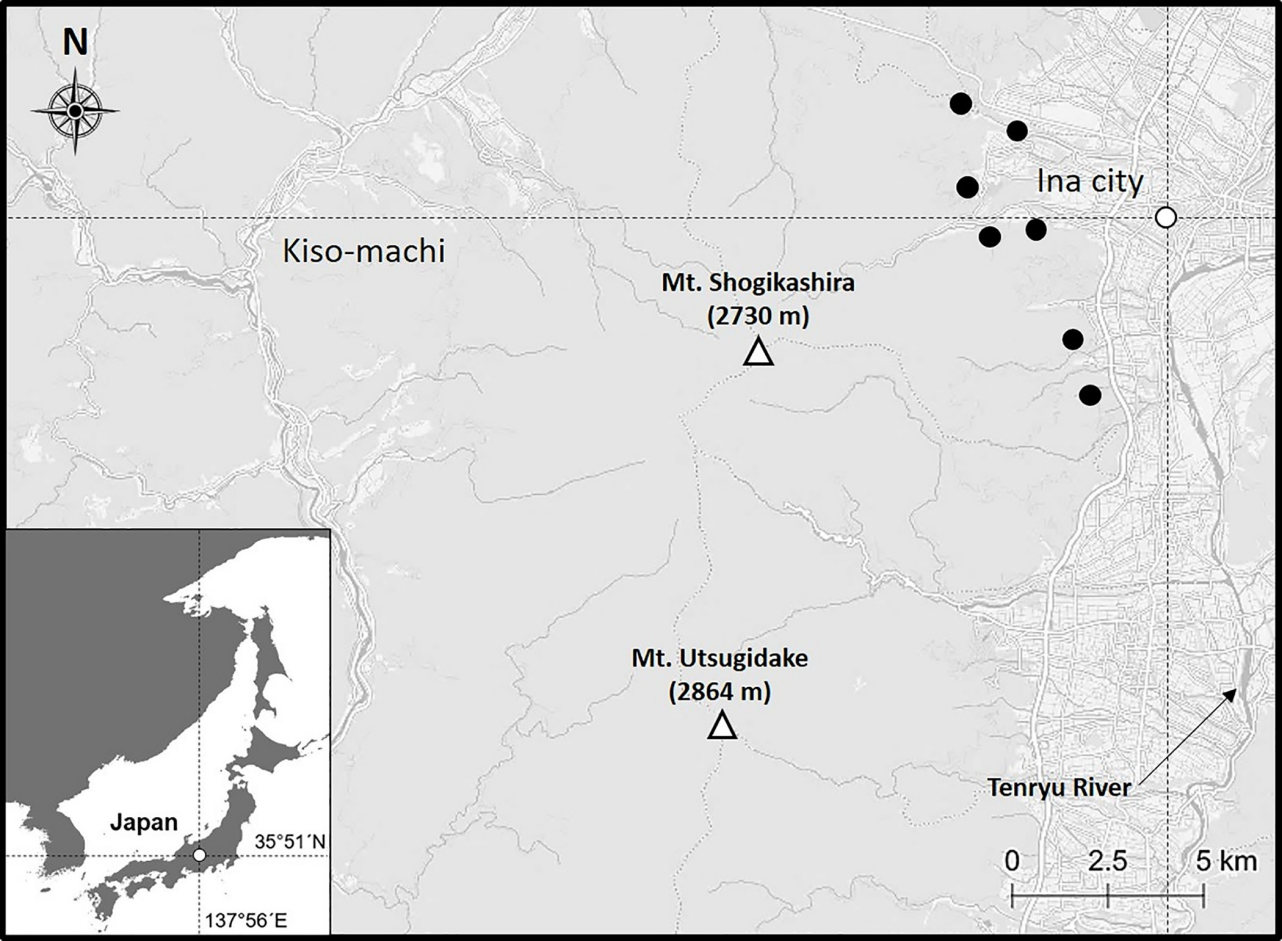
Study area in Nagano Prefecture, Japan. Black circles indicate the capture site of the GPS-collared Asiatic black bears.

The study was mainly conducted in the northeastern part of the Central Japan Alps, with the Ina Valley spreading to the east and the Kiso Valley, in the southwest of the Nagano Prefecture (35° 51´ N, 137° 56´ E), Japan (Fig 1). Altitude ranged from 590 to 2956 m, with a steep terrain in the mountains. The landscape of the foothills comprised residential areas, dense roads, and agricultural lands, such as corn and paddy field. Vegetation in this area varied with altitude, with dogwood (*Cornus controversa*) and Japanese walnut (*Juglans mandshurica*) being part of the streamside forest (<700 m). Mountain zone (800–1600 m) was dominated by Larch (*Larix leptolepis*)- and Japanese red pine (*Pinus densiflora*) plantation. This altitude range was also partly dominated by Mongolian oak (*Quercus crispula*), konara oak (*Q*. *serrata*), chestnut (*Castanea crenata*), and *Prunus* spp.. Subalpine zone (>1600 m) was dominated by Russian rock birch (*Betula ermanii*) and Maries’ fir (*Abies mariesii*). The weather is cool in summer and cold in winter; the mean annual temperature is 11.8°C and the total annual precipitation is 1497 mm [21]. Survey periods could be divided into four season depending on plant phenology of the study area: spring (April–May; leaf unfolding), early-summer (June–July; some berries, such as ripe *Prunus* spp. and *Rubus* spp.), late-summer (August–September; many type of ripe berries), and autumn (October–November; ripe hard mast, such as acorns and nuts).

### Asiatic black bear capture and location data retrieval

During spring and summer in 2016–2018, we attached GPS collars (VECTRONIC Aerospace GmbH, Berlin, Germany) to 15 adult (age ≥ 5 years) Asiatic black bears (7 males, 8 females). Local hunters set barrel traps for capturing bears as well as other traps to capture wild boar (*Sus scrofa*) or Japanese macaque (*Macaca fuscata*) at the foot of Mt. Shogikashira (Fig 1) for managing purposes. We captured bears which were mistakenly attracted by the traps set for the wild boar or Japanese macaque as well as the ones which were caught in the barrel traps. We immobilized the bears with a mixture of ketamine (10mg/kg) and xylazine (1mg/kg) hydrochloride as a tranquilizer using dart gun. We weighed their body mass with a digital handling scale, extracted an upper premolar tooth for age estimation, and then attached a GPS collar with a drop-off device to each bear. The series of protocol, such as immobilization and collaring were conducted by specifically trained and certified scientists. We tried to keep the bears on a relatively flat ground to avoid occlusion of the trachea during handling. The sedation time was kept to the minimum, usually no longer than 1 h. We programmed all collars to collect fixes at 1-h intervals from April to November, except for one collar (2 h intervals). We downloaded the data using remote communication in the field or via satellite communication.

### Cluster identification and scat sample collection

Between April and November for each year from 2017–2018 excluding period of bear hibernation from December to March, we conducted field surveys to collect the bears’ scat samples at consecutive GPS clusters. A GPS cluster was defined as locations where the Asiatic black bears spend a minimum of three fixes (i.e., <3–6 h) within the same area (<20 m radius). The location data of each bear were displayed using QGIS software [22]. The center coordinates of GPS cluster were programmed into handheld GPS units, and we visited the GPS clusters within 5 days after data acquisition and carefully searched for scats in a 20 m radius to account for GPS collar error (6.7±5.7 m [mean ± SD]; A. Takii, unpublished data). We randomly selected 2–4 GPS clusters at different day points and visited them; this procedure was performed twice a month for each bear (therefore, 4–8 GPS clusters /month for each bear). However, because of several difficulties such as collar malfunction, satellite communication errors, and labor constraints, we were unable to collect scat samples of all individuals equally throughout the survey period.

Once we found a scat, we recorded the estimate age of the scat, date of collection, GPS location, and then stored it at −20°C until further analysis. Scat age was determined based on color, moisture in relation to weather conditions, and remaining odor level. One female (F04) was accompanied by a cub in 2017, so we distinguished adult and cub scats by size and collected only the adult’s scat. Although Asiatic black bears are primarily solitary, during the mating season from June to July, they sometimes congregate in a small area (<50 m radius) with low activity levels for several days [23,24] [T. Mori, personal observation] similar to grizzly bears [25]. Therefore, we did not collect scat samples at GPS clusters where the bears being tracked stayed for >48 h within <50 m during the mating period. In addition, to avoid collecting scat samples of Asiatic black bears not being tracked, we collected scat samples only if the estimated scat deposit dates matched the days on which the collared Asiatic black bears stayed in the location.

In 2017, we surveyed at least 52 GPS clusters and collected 105 scat samples from April to October. In 2018, we surveyed 225 GPS clusters and collected 354 scat samples from 123 of those GPS clusters (54.6%) from April to November. We also collected seven scats from three captured bears during capturing in 2017. The total number of scat samples collected (*N*) in 2017 and 2018 was 105 and 361 (7 + 354), respectively. We sometimes found multiple scats at the same cluster (particularly in autumn) and collected them all; because gut retention times of Asiatic black bears vary greatly (range: 3–44 h) depending on their physical condition and diets [26], it was a possibility that scats collected at the same cluster can be derived from independent feeding events even if the scat contents are the same. However, excessive sampling of scats at the same cluster may cause autocorrelation and overrepresentation of certain food items. Thus, we used up to five scats (randomly chosen) for subsequent analysis when collecting five or more scats at the same cluster to correct this bias [27]. Eventually, we used 105 and 356 scats for the analysis in 2017 and 2018, respectively.

### Scat analysis

Before scat analysis, individual food items were extracted from each scat sample by carefully washing the sample with water in a sump while passing the sample through two sieves (2.0 and 1.0 mm in diameter) until the water was clear. The food items on the sieves were identified to the lowest-possible taxon. It was difficult to distinguish several species, so we combined the species as follows: Mongolian oak, konara oak, sawtooth oak (*Q*. *acutissima*), and chestnut under the Fagaceae family, and dogwood and large-leaf dogwood (*Cornus macrophylla*) under *Cornus* spp.

We used two methods of evaluating the contents of the scat samples according to percent volume:

- In 2017, we separated the food items from one another and visually estimated the percent volume [28] using a 5-point scale: <1%, 1–25%, 25–50%, 50–75%, and 75–100%.
- In 2018, we estimated the percent volume using the point-frame method [29] to reduce the time and effort required.

It has been proven that the two methods yield similar results [29]. The percent volumes estimated using both methods were reassigned to 1%, 12.5%, 37.5%, 62.5%, and 87.5%. The dietary composition was expressed as percent important value (%IV, [22]) and was calculated step by step using the percent volume as follows:

$$\%V_{i}=\sum iR/N,$$

where %*V*_*i*_ is the mean percentage volume of food item *i*, *R* is the percent volume of food item *i* in a scat sample, and *N* is the total number of scat samples.

$$\%F_{i}=(\mathrm{Number}\;\mathrm{of}\;\mathrm{scat}\;\mathrm{samples}\;\mathrm{with}\;\mathrm{food}\;\mathrm{item}\;i/N)\times 100,$$

where %*F*_*i*_ is the percent frequency of occurrence.

$${\mathrm{IV}}_{i}=(\%V_{i}\times \%F_{i})/100,$$

where IV_*i*_ is the important value.

$$\%{\mathrm{IV}}_{i}=({\mathrm{IV}}_{i}/\sum \mathrm{IV})\times 100,$$

where %IV_*i*_ is the percent important value.

### Data analysis

To evaluate the degree of dietary specialization in Asiatic black bears, we calculated the proportion similarity index (PSI) using the program IndSpec1 [30]. The PSI shows the overlap between the diet of one individual and the diet of the entire population as follows:

$$\mathrm{PSI}=1.0-0.5\sum_{j}|p_{ij}-q_{j}|=\sum_{j}\min(p_{ij},q_{j}),$$

where *p*_*ij*_ are the proportion of %IV of food item *j* in individual *i*’s diets and *q*_*j*_ are the proportion of %IV of food item *j* in the entire population (pooled scat samples). The PSI was calculated for each Asiatic black bear being tracked and summarized as a population-wide measure of dietary specialization for a season. It ranged from 0 (completely overlapping) to 1 (completely different). These indices were calculated only for individuals for whom three or more scat samples were collected.

To examine the seasonal diet breadth of each Asiatic black bear being tracked, we also calculated the Shannon–Wiener diversity index (*H'*) on the basis of %IV of food items (green vegetation, 12 fleshy fruits, two seeds, insects, mammals, and crops). These indices also were calculated only for individuals for whom three or more scat samples were collected.

To test differences in dietary specialization on the basis of sex, season, and years, we used a generalized linear mixed effect model (GLMM) with binomial error distribution because response values were proportional values. The response variable was the monthly PSI of each individual in 2017–2018, and explanatory variables were the sex (male, female), season (spring, early-summer, late-summer, and autumn), and interactions between these values. In addition, we used individual ID as a random factor in the model. The forward stepwise selection with Akaike information criteria (AIC) were employed to evaluate the fit of the models and the relative importance of the explanatory variables. We considered models with ΔAIC<2 as equivalent support [31]. We also tested for differences in the degree of dietary diversity on the basis of sex, season, and years using the monthly *H*’ in same manner but using Gamma distribution error because *H*’ values were continuous variable of zero or more. These statistics were calculated using package “lme4” [32] in R. 3.5.1 [33].

## Results

### Data

The average tracking duration of the 15 Asiatic black bears during the study period (except during hibernation) was 2–13 months, with a mean of 6.2 months. Refer to Table 1 for details of the GPS-collared Asiatic black bears.

**Table 1:** List of GPS collared Asiatic black bears and their sex, age, body mass, and sampling periods in Nagano Prefecture, Japan (2017–2018).

ID	Sex	Age	Body mass	2017				2018			
				Spring	Early summer	Late summer	Autumn	Spring	Early summer	Late summer	Autumn
M01	M	8	60kg	○	●						
M02	M	6	41kg	○	●				○	○	○
M03	M	6	51kg			○	●				
M04	M	12	82kg			○	○	○			
M05	M	17^a^	78kg					○	○	○	○
M06	M	7	53kg						○	○	○
M07	M	18	116kg						●	○	○
F01	F	14^a^	55kg	○	○					○	
F02	F	5	35kg		○	○	○	○	○	○	○
F03	F	23	45kg		●	○	○				
F04	F	15	36kg			○	○	○	○	○	○
F05	F	14	45kg			○	●	○			
F06	F	5	39kg		●	●	○		●	○	○
F07	F	10	52kg						○	○	○
F08	F	8	42kg							○	○
Number of male bears				2	0	2	1	2	3	4	4
Number of female bears				1	2	4	4	3	3	6	5
Total number of bears				3	2	6	5	5	6	10	9

White circles indicate normally operated collars and that we could collect bears' scats; black circles indicate normally operated collars but we were unable to collect any bears' scats because of several difficulties such as collar malfunction, satellite communication error, and labor constrains.

^a^indicate estimated ages because we were unable to determinate the exact age.

### Dietary specialization and diet diversity

**Fig 2:**
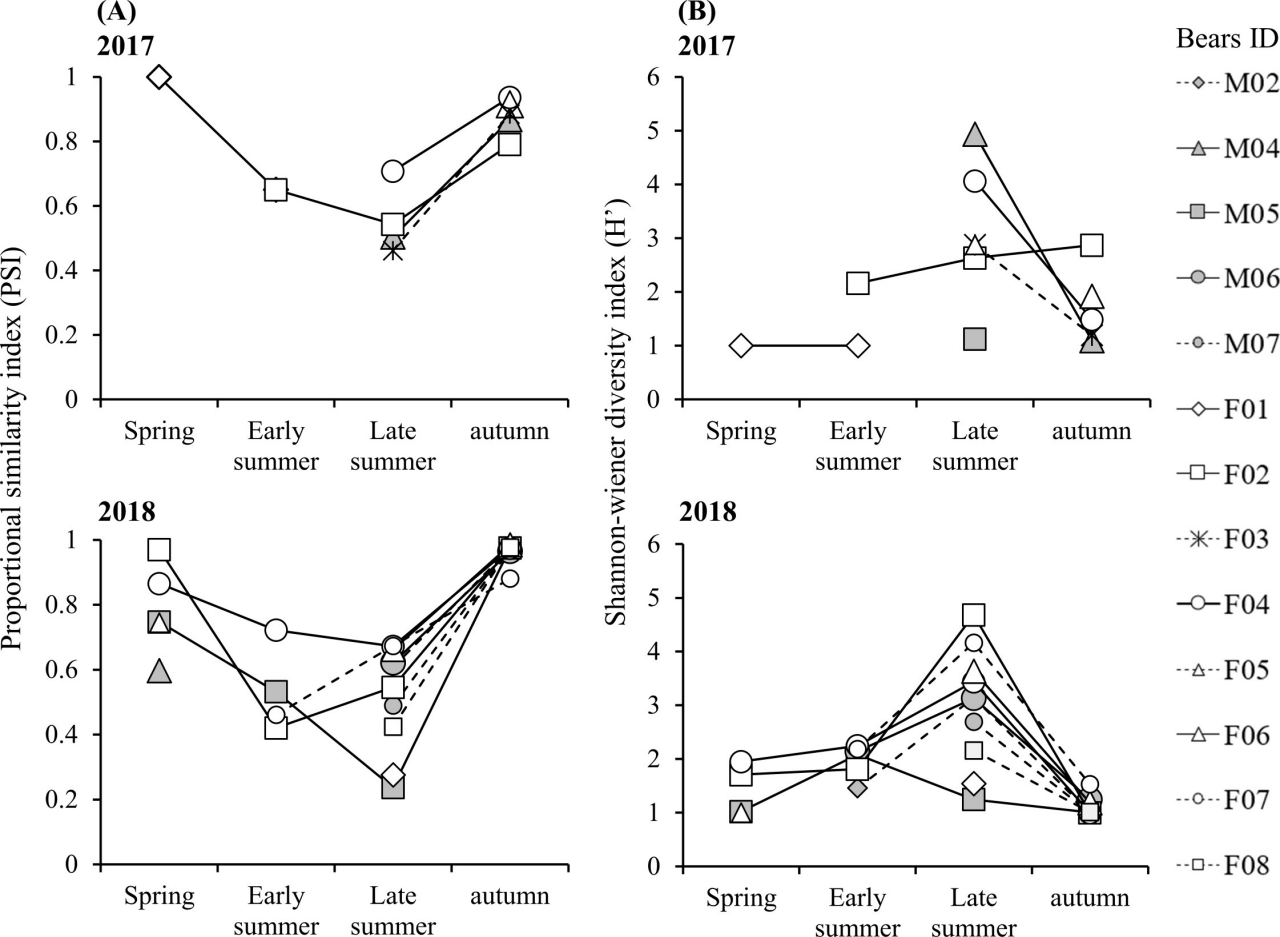
**(A) PSI and (B) Shannon–Wiener diversity index (H’) for foods used by Asiatic black bears in Nagano Prefecture, Japan from 2017–2018.** PSI, proportion similarity index.

The PSI of each Asiatic black bear ranged from 0.24 to 1.00 throughout the study. From the analysis of GLMM, three candidate models (ΔAIC < 2) with higher probability of explaining the data were selected (Table 2). All three models included seasons as explanatory variables, with PSI values lower in early- and late-summers than autumn (with significant or significant trends) (Fig 2A; Table 2).

**Table 2:** Results of the model selection for PSI (A) and (B) Shannon–Wiener diversity index (H’) based on sex, season, and year. The candidate models (ΔAIC < 2) were ordered according to Akaike information criteria (AIC).

**(A) Proportional similarity index**			
**Model**	**No. 1** **(Season)**	**No. 2** **(Season+Sex)**	**No. 3** **(Season+Year)**
	Estimate (SE)	Estimate (SE)	Estimate (SE)
Intercept	2.65 (1.08)^a^	2.74 (1.13)^a^	2.78 (1.22)^a^
Season (ref: autumn)			
spring	–0.95 (1.50)	–0.93 (1.51)	–0.93 (1.50)
early-summer	–2.36 (1.36)^b^	–2.41 (1.37)^b^	–2.36 (1.36)^b^
late-summer	–2.53 (1.20)^a^	–2.54 (1.20)^a^	–2.52 (1.20)^a^
Sex (ref: male)		–0.24 (0.83)	
Year (ref: 2018)			–0.20 (0.85)
df	4	5	5
AIC	13.41	15.33	15.35
ΔAIC	0.00	1.92	1.94
**(B) Shannon–Wiener index**			
**Model**	**No. 1** **(Season)**	**No. 2** **(Season+Year)**	**No. 3** **(Season+Sex)**

	Estimate (SE)	Estimate (SE)	Estimate (SE)
Intercept	0.15 (0.11)	0.23 (0.12)^b^	0.15 (0.14)
Season (ref: autumn)			
spring	0.13 (0.12)	0.16 (0.12)	0.13 (0.12)
early-summer	0.40 (0.13)^a^	0.42 (0.12)^a^	0.40 (0.13)^a^
late-summer	0.89 (0.09)^a^	0.91 (0.09)^a^	0.89 (0.09)^a^
Sex (ref: male)			–0.01 (0.19)
Year (ref: 2018)		–0.13 (0.09)	
df	4	5	5
AIC	74.23	74.43	76.22
ΔAIC	0.00	0.20	1.99

^a^indicates significant at P < 0.05

^b^indicates significant trends at 0.05 < P < 0.1

The Shannon–Wiener diversity index (*H'*) of each Asiatic black bear ranged from 1.00 to 4.93 throughout the study. Three candidate models were selected (Table 2). All three models included seasons as explanatory variables, with *H*’ values higher in early- and late-summers than autumn (with significant) (Fig 2B; Table 2). In particular, *H*’ values were highest in late-summer.

### Dietary composition

**Fig 3:**
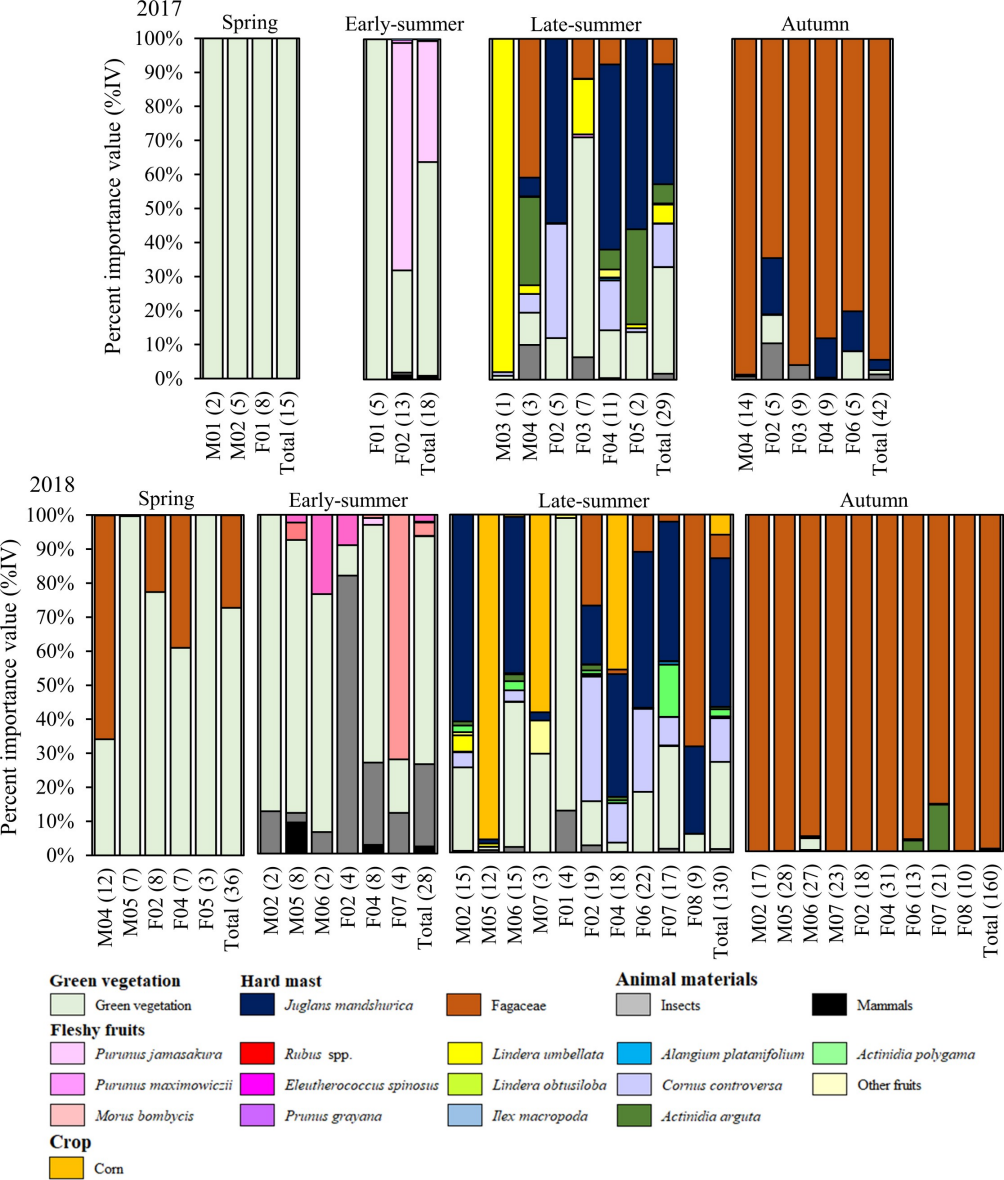
Seasonal dietary composition of 15 Asiatic black bears in Nagano Prefecture, Japan (2017–2018). The numbers in parentheses on the x axis show the number of scat samples for each bear.

The Asiatic black bears in our study area consumed green vegetation and overwintered seeds of the Fagaceae family in spring; green vegetation, animal matters, and fleshy fruits in early-summer; green vegetation, many types of fleshy fruits, seed of the Fagaceae, and crops in late-summer; the fleshy fruits and seed of the Fagaceae family in autumn (Fig 3). Dietary composition varied among individuals from spring to late-summer, but did not vary in autumn when all individuals heavily depended on the Fagaceae family (Fig 3).

The dietary composition between sexes showed a similar trend in spring and autumn, with green vegetation and the Fagaceae family constituting a high proportion of the diet, respectively (Table 3). It was difficult to interpret sex-based dietary differences in early- and late-summer because of low sample sizes (2**–**10 bears per season). However, we did find a sex-based dietary differences in late-summer 2018: 6 females consumed seeds of Fagaceae family to some extent (~18.3 ± 24.3%IV), but 4 males did not (0.0 ± 0.0%IV) (Table 3). Dietary composition also varied greatly within sexes in early- and late-summer.

**Table 3:** Seasonal and sexual variations in dietary composition determined from six major food categories for Asiatic black bears in Nagano Prefecture, Japan (2017–2018).

		Green vegetation	Fleshy fruits	Hard mast (Japanese walnut)	Hard mast (Fagaceae)	Animal materials	Crops
**2017**							
Spring	M(2)	100.0 ± 0.0	0.0 ± 0.0	0.0 ± 0.0	0.0 ± 0.0	0.0 ± 0.0	0.0 ± 0.0
	F (1)	100.0	0.0	0.0	0.0	0.0	0.0
Early-summer	M (0)	–	–	–	–	–	–
	F (2)	65.0 ± 35.0	34.0 ± 34.0	0.0 ± 0.0	0.0 ± 0.0	1.0 ± 1.0	0.0 ± 0.0
Late-summer	M (2)	5.3 ± 4.2	66.5 ± 32.4	2.7 ± 2.7	20.4 ± 20.4	5.1 ± 5.1	0.0 ± 0.0
	F (4)	26.2 ± 22.1	26.2 ± 6.3	41.1 ± 23.7	4.8 ± 5.0	1.8 ± 2.8	0.0 ± 0.0
Autumn	M (1)	0.5	0.0	0.1	98.5	0.9	0.0
	F (4)	4.2 ± 4.1	0.1± 0.1	9.9 ± 6.1	82.0 ± 11.6	3.8 ± 4.4	0.0 ± 0.0
**2018**							
Spring	M (2)	66.9 ± 32.8	0.0 ± 0.0	0.0 ± 0.0	33.0 ± 32.7	0.1 ± 0.0	0.0 ± 0.0
	F (3)	79.5 ± 16.0	0.0 ± 0.0	0.0 ± 0.0	20.5 ± 16.0	0.0 ± 0.0	0.0 ± 0.0
Early-summer	M (3)	79.4 ± 7.1	10.3 ± 9.8	0.0 ± 0.0	0.0 ± 0.0	10.3 ± 2.8	0.0 ± 0.0
	F (3)	31.7 ± 27.4	28.0 ± 31.3	0.0 ± 0.0	0.0 ± 0.0	40.3 ± 30.1	0.0 ± 0.0
Late-summer	M (4)	24.4 ± 15.1	8.2 ± 4.6	27.8 ± 26.5	0.0 ± 0.0	0.7 ± 0.6	38.8 ± 40.7
	F (6)	26.0 ± 28.5	17.5 ± 14.3	27.8 ± 15.7	18.3 ± 24.3	2.6 ± 4.4	7.7 ± 17.1
Autumn	M (4)	0.9 ± 1.5	0.1 ± 0.2	0.0 ± 0.1	98.9 ± 1.9	0.1 ± 0.2	0.0 ± 0.0
	F (5)	0.1 ± 0.1	3.4 ± 5.3	0.1 ± 0.2	96.4 ± 5.4	0.0 ± 0.0	0.0 ± 0.0

Mean values±standard deviations are arranged.

F; female, M; male.

Numbers in parentheses are the number of bears per category.

## Discussion

Scat sampling, combined with GPS technologies, enabled us to conduct surveys of the individual food habits of Asiatic black bears. Although seasonal changes in the bears’ dietary composition were observed at the population level, the dietary components differed among individuals in most seasons (Fig 3). The extent of dietary specialization seasonally changed, and sex-specific differences in diet were observed. To the best of our knowledge, this is the first study reporting individual variation in diet throughout the year in Asiatic black bears. We confirmed that dietary specialization is a universal phenomenon in Asiatic black bears as well as other bear species (e.g., polar bears (*U*. *maritimus*) [34], grizzly bears (*U*. *arctos*) [35], and American black bears (*U*. *americanus*) [36]).

### Dietary specialization in relation to ecological context

Foraging strategy of free-ranging animals is complex, and each individual need to meet their nutritional goals in relation to their intrinsic traits (sex- and age differences, social ranks, and experience) by selecting optimal foods in a fluctuating environment [6]. In the environment where food abundance is limited, dietary specialization may increase owing to intraspecific competition [3,7,37] in addition to sex- and age-based differences. As expected, in this study, dietary specialization in Asiatic black bears was high in summer and low in spring and autumn in both sexes, indicating that food abundance is an important driver of seasonal dietary specialization in Asiatic black bears. Simultaneous leaf unfolding and fruiting seeds of the Fagaceae family supply moderate or abundant foods for Asiatic black bears in spring and autumn, respectively, resulting in low dietary specialization. In contrast, low-nutritional green vegetation with high fiber content [38] and slightly fleshy fruits are available in summer, that is, food is scarce (especially in July), resulting in high dietary specialization (Figs 2 and 3).

In addition, this foraging adjustment among individuals might be regulated by potential food diversity in the environment [4]. For example, tetra fish (*Astyanax lacustris*) in Pantanal wetlands experience strong intraspecific competition in the dry season, but dietary specialization is low because of fewer resources available, which constrains the diversification of dietary composition [39]. The spring diet of Asiatic black bears comprises only green vegetation and overwintered seeds of the Fagaceae family, if available [8], resulting in low dietary specialization because of food diversity constrains (Figs 2 and 3). In contrast, in summer, food diversity is higher compared to other seasons, but abundance is lower [14]. In our study, individuals extended their diet breadth to include a variety of foods, such as green vegetation, fruits, and insects in summer (Fig 3). Because Asiatic black bears forage on optimal foods from green vegetation, fleshy fruits, and insects according to age- and sex-based nutritional requirements under high intraspecific competition (e.g., social dominance), it results in high dietary specialization and dietary diversity. Furthermore, brown bears maximize energy intake by consuming a variety of food to optimize their macronutrient ratios, in which protein comprised approximately 20% of the digestible energy in the diets [40]. It is likely that Asiatic black bears follow mixed diet strategy to maximize energy intake in summer when food resources are scarce but insects (high protein) are available.

Unfortunately, we did not determine actual food abundance and diversity of bears in our study. However, the body condition of captured Asiatic black bears in our study area changed with the fluctuation of seasonality of general food abundance and diversity. The body condition of bears was lowest in early-summer and late-summer (resources less abundant) and highest in autumn (resources more abundant) [S. Izumiyama, personal observation], suggesting that intraspecific competition was greater in summer in our study area. Thus, we believed that dietary specialization in Asiatic black bears changes seasonally in relation to interactions between food abundance and diversity.

Ripening fruit availability peaks from late-summer to autumn, offering Asiatic black bears the opportunity to consume many types of fruits in autumn [41,42]. In this study, however, all bears heavily depended on seeds of the Fagaceae family from autumn (Fig 3), although other fruits (e.g., Japanese walnut, tara vine, and dogwood) were available [T. Mori, personal observation]. Since Asiatic black bears physiologically enter a hyperphagic phase in preparation for hibernation in late-autumn [43], they need to consume a huge amount of high-quality foods. The seeds of the Fagaceae family contain higher concentration of carbohydrates and lipids compared to fleshy fruits [8,44], and these are the dominant species in broad-leaved forests [14]. Asiatic black bears can obtain a large amount of energy from seeds of the Fagaceae family, except in years with extremely low seed production [45]. Therefore, seeds of the Fagaceae family seem to play a more important role compared to fleshy fruits in fat accumulation before hibernation in Asiatic black bears, so no dietary specialization occurred in autumn.

### Dietary specialization in relation to sexual differences

Individual characteristics, such as sex, age-related morphological differences, and reproductive status, are major contributors to dietary specialization in the Ursidae family. For example, because of their hunting ability, adult male polar bears tend to target bearded seals (*Erignathus barbatus*), the largest northern seals, compared to female and subadult polar bears [34]. American black bears with yearlings tend to forage for blueberries, whereas lone bears forage for ants, probably because of differences in physiological requirements [36]. We found it difficult to investigate sex-based dietary differences because of the small sample size. However, we believe that our results suggested the possibility of sex-based dietary differences.

In 2018, all females consumed the seeds of Fagaceae earlier than males (male from autumn and females from late-summer). The difference in the duration of hibernation between sexes might be one reason. In general, the timing of den entry is earlier and the duration of hibernation longer in females compared with males [46–48]. Because longer hibernation results in higher body mass loss [49], females need to accumulate more fat earlier than males. In addition, females need to accumulate sufficient fat to get pregnant and for lactation [20]. Thus, differences in energy requirements during hibernation and pregnancy might result in sexual differences in the timing of feeding on the Fagaceae family in Asiatic black bears.

Furthermore, in this study, we confirmed that diet composition differed greatly within sexes. This result indicated that individuals' age, social rank, and experience besides sex-based differences may also affect dietary specialization.

## Future study and conclusion

Because the Asiatic black bears’ diet shows considerable annual variation in relation to fruit production [11,12,50], our results are just a “snapshot” of the dietary specialization traits. In our study, based on the data of 2 years survey, we concluded that most bears depend on seeds of the Fagaceae family during autumn. However, bears in Toyama Prefecture show individual variation in their diets even in autumn during poor-masting years [13]; some bears foraged on agricultural crops or animal materials rather than the seeds of Fagaceae in autumn. Dietary specialization may also occur in autumn when the production of Fagaceae is scarce.

This study had some limitations. First, the monthly sample size was small (2–10 bears), so we were unable to determine how individuals’ traits other than sexual differences (e.g., age, experience, and social rank) contribute to dietary variation within sexes. Second, scat samples per individual were limited, especially in early-summer. Third, we could not determine the actual food abundance and diversity of bears. Therefore, the results of this study must be interpreted with caution, and it is necessary to increase the number of samples and evaluate food abundance to corroborate these results. Further studies are needed in order to determine the relationship between dietary specialization and factors other than sexual differences.

In this study, we provided evidence for dietary specialization in the food habits of Asiatic black bears. The level of dietary specialization seasonally changed depending on food abundance, food diversity, and sex-based differences. However, dietary specialization in the Asiatic black bears involved multiple factors other than sexual differences.
